# Illness perception and health care use in individuals with irritable bowel syndrome: results from an online survey

**DOI:** 10.1186/s12875-021-01499-5

**Published:** 2021-07-19

**Authors:** Juliane Schwille-Kiuntke, Solveig Lu Rüdlin, Florian Junne, Paul Enck, Katja Brenk-Franz, Stephan Zipfel, Monika A. Rieger

**Affiliations:** 1 Institute of Occupational and Social Medicine and Health Services Research, University Hospital Tübingen, Wilhelmstraße 27, 72074 Tübingen, Germany; 2 Department of Internal Medicine VI: Psychosomatic Medicine and Psychotherapy, University Hospital Tübingen, Tübingen, Germany; 3 Department for Psychiatry and Psychotherapy, Clinic for Psychosomatic Medicine Ginsterhof, Rosengarten, Germany; 4 Department for Psychosomatic Medicine and Psychotherapy, Otto Von Guericke University Magdeburg, Magdeburg, Germany; 5 Institute of Psychosocial Medicine, Psychotherapy and Psychooncology, Jena University Hospital, Jena, Germany

**Keywords:** Illness perception, Health care use, Irritable bowel syndrome, Health Services Research

## Abstract

**Background:**

Individual illness perception is known to influence a range of outcome variables. However, little is known regarding illness perception in irritable bowel syndrome (IBS) and its relation to the use of the health care system. This study hypothesised a relationship between illness perception and inappropriate health care use (under-, over- and misuse).

**Methods:**

An internet-based, cross-sectional study in participants affected by IBS symptoms was carried out (April – October 2019) using open questions as well as validated standardized instruments, e.g. the illness perception questionnaire revised (IPQ-R) and its subscales. Sub-group comparisons were done non-parametrically and effect sizes were reported. Potential predictors of (1) conventional health care utilisation and (2) utilisation of treatment approaches with lacking or weak evidence regarding effectiveness in IBS were examined with logistic regression analyses and reported as odds ratio (OR) and 95% confidence interval.

**Results:**

Data from 513 individuals were available. More than one-third (35.7%) of participants were classified as high utilisers (> 5 doctor visits during the last year). Several indicators of inappropriate health care use were detected, such as a low proportion of state-of-the-art gynaecological evaluation of symptoms (35.0% of women) and a high proportion of individuals taking ineffective and not recommended non-steroidal antirheumatic drugs for IBS (29.4%). A majority (57.7%) used treatment approaches with lacking or weak evidence regarding the effectiveness in IBS (e.g. homeopathy). Being a high utiliser as defined above was predicted by the perceived daily life consequences of IBS (IPQ-R subscale “consequences”, OR = 1.189 [1.100–1.284], p ≤ 0.001) and age (OR = 0.980 [0.962–0.998], p = 0.027). The use of treatment approaches with lacking or weak evidence was forecasted by the perceived daily life consequences (OR = 1.155 [1.091–1.223], p ≤ 0.001) and gender (reference category male: OR = 0.537 [0.327–0.881], p = 0.014), however effect sizes were small.

**Conclusions:**

Daily life consequences, perceived cure and personal control as aspects of individual disease perception seem to be related to individuals’ health care use. These aspects should be a standard part of the medical interview and actively explored. To face inappropriate health care use patients and professionals need to be trained. Interdisciplinary collaborative care may contribute to enhanced quality of medical supply in IBS.

**Supplementary Information:**

The online version contains supplementary material available at 10.1186/s12875-021-01499-5.

## Background

Irritable bowel syndrome (IBS) is a common gastrointestinal disorder and causes considerable individual burden, restrictions in quality of life and high direct and indirect costs for the society [1]. Affected patients suffer from abdominal pain and discomfort, as well as changes in stool frequency and consistency, such as diarrhoea and constipation [1]. In former times, the standard dogma was, that in IBS no somatic correlates or changes exist, which are detectable with clinical routine methods. This led to the term “functional disease” [1]. However, during the last two decades, there is profound evidence for changes in gut permeability, motility and sensitivity, as well as multidirectional interactions between gut, brain and microbiome as pathogenic components of IBS [1]. A high degree of comorbidity with further “functional” (e.g. dyspepsia in > 20% [2], fibromyalgia > 30% [2], chronic pelvic pain > 50% [2]), as well as psychiatric diagnoses (e.g. depression, anxiety in ~ 50% [1]) is well established. The high proportion of patients suffering from IBS and at least one further diagnosis, as well as a dysfunctional illness representation may contribute to the high degree of health care utilisation, which is reported to be associated with IBS [3].

### Health care use in IBS

Research about the utilisation of the health care system is often based on the Andersen model [4]. This is a conceptual framework which distinguishes three types of factors: the need, enabling factors (e.g. health insurance, social or family support), and predisposing factors for health care use (e.g. age, gender, educational level or motivation) [4, 5].

Data related to health care use in individuals suffering from IBS are conflicting: Some studies found symptom severity and duration predicting the utilisation of health professionals’ advice [6], others reported psychological features as key factors [7], as well as socioeconomic factors such as educational level and insurance status [8]. To the best of our knowledge, there is no coherent model comprehensively explaining healthcare use in IBS, but a variety of different aspects are known which influence health care seeking behaviour [8].

The term “inappropriate health care” can be conceptualised in different ways, one of which is in the classification of the United States National Roundtable on Health Care Quality [9]. In this consensus statement three health quality problems were distinguished: (i) underuse, defined as “the failure to provide a health care service when it would have produced a favourable outcome for a patient” (ii), overuse, which “occurs when a health care service is provided under circumstances in which its potential for harm exceeds the possible benefit”, and (iii) misuse, which is the case “when an appropriate service has been selected but a preventable complication occurs and the patient does not receive the full potential benefit of the service” [9]. The distinction of over- and misuse may be blurred, for example in the case of a subjective, but no objective need for a medical service with usually proven benefit [10]. To the best of our knowledge, this concept of inappropriate health care use has never been applied to data from an IBS affected population.

In daily clinical routine, the medical support of individuals suffering from IBS is frequently characterised by discontinuous treatment pathways and inappropriate health care. Clinical courses with a high number of investigations (sometimes repeatedly) and multiple pharmacological and non-pharmacological treatment attempts are often seen. At the same time, a substantial proportion of approximately 20% of IBS patients never seek medical advice [11].

### The concept of illness representation

The individual illness representation is the sum of cognitions and beliefs of patients about their illness. This definition dates back to the Common Sense Model of illness representation developed by Leventhal and colleagues in the 1980’s [12]. They distinguish emotional and cognitive representations, the latter including beliefs about causes, consequences, timeline, and controllability of the condition [12]. In some publications the term illness perception means one dimension of illness representation (among others) [13], but others [14] used illness perception and illness representation as a synonym – just like we will do in this paper.

Based on several studies, a causal relationship between illness perception and clinical outcomes such as quality of life [15] and symptom severity [16] seems likely, which allows a distinction between rather beneficial and dysfunctional illness representations.

The importance of the illness perception was shown regarding mental diseases [17], as well as somatic diseases, such as chronic obstructive pulmonary disease [18], myocardial infarction [19], and breast cancer [20]. For example high attendance at rehabilitation is predicted – among others – by feelings of controllability of the disease in patients after myocardial infarction [19]. And in patients suffering from chronic obstructive pulmonary disease an association of illness perception, quality of life and coping behaviour was found [18] – which shows that illness perception is related to patients’ compliance and to relevant patient reported outcomes.

There are only a few studies investigating health care use and individual perceptions of IBS: For example, van Dulmen et al. [21] reported somatic attribution of symptoms increasing the probability of further health care seeking in primary care despite exclusion of a somatic origin. And Gudleski et al. [8] found higher sense of responsibility for managing IBS symptoms in patients who showed higher levels of internal locus of control beliefs.

### Research questions and hypotheses

Based on these considerations, we considered individual illness perceptions as a predisposing factor according to Andersen [4] and addressed the following questions in an explorative approach:

Question 1: Which conventional health services and treatment approaches with lacking or weak evidence regarding effectiveness in IBS treatment were used by individuals suffering from self-declared IBS symptoms?

Question 2: Are there differences in the individual health perception between subgroups of individuals who use the health care system in different ways? These subgroups are built on the basis of the following criteria:

i) normal versus (vs.) high utilisers of conventional medicine (> 5 doctor visits during the last year).
ii) users vs. non-users of IBS-related medication.
iii) users vs. non-users of treatment approaches with lacking or weak evidence regarding effectiveness in IBS.

Question 3: Are the frequency of doctor visits and the use of treatment approaches with weak or lack of evidence regarding effectiveness in IBS predictable by individual illness perception, demographic (age, gender) and mood variables (symptoms of depression and anxiety disorder)?

We postulated a substantial degree of inappropriate health care use, mainly underuse. Furthermore, it is hypothesized, that a more intensive health care use (of conventional medicine as well as of treatment approaches with lacking or weak evidence) is associated with a more dysfunctional illness perception, and elevated levels of depression and anxiety.

## Methods

The presented study was designed as an internet-based, cross-sectional survey, which was carried out between April 2019 and October 2019.

The proposal of the study was reviewed and approved by the Ethical Committee of the Tübingen University Hospital and Medical Faculty (Number 763/2015BO2 in 2015/16 and Addendum in 2018/19). Participants were asked to provide consent for the use of their completed questionnaire data for research purposes by ticking an extra “consent box” in the online questionnaire, but remained anonymous.

### Data collection

Our sample consists of people who indicated having received the diagnosis of IBS. The inclusion was based on self-declaration only. Rome IV criteria as diagnostic gold standard [22] were additionally assessed to validate the diagnosis as stated below. Furthermore, a minimum age of 18 years (self-declared) was requested for participation. The data collection was performed using UniPark/Questback package (www.unipark.com). Potential participants were recruited via an invitation email sent to every enrolled student at Tübingen University and to all employees of Tübingen University and Tübingen University Hospital. Additionally, the invitation was distributed via a national self-aid group for IBS (Deutsche Reizdarmselbsthilfe e.V., www.reizdarmselbsthilfe.de, last access 29^th^ May 2021) and the patient forum of the German Society of Neurogastroenterology and Motility (Unabhängiges Informationsforum für Magen-Darm-Erkrankungen der Deutschen Gesellschaft für Neurogastroenterologie – MAGDA, magendarm-forum.de, last access 29^th^ May 2021) using mailing lists and websites. Flyers with QR code to the questionnaire were placed in doctor’s offices of gastroenterologists, family practitioners, and nutrition therapists in Hamburg, Tübingen, and Jena, Germany.

### The questionnaire

The two key elements of our questionnaire were the revised version of the validated illness perception questionnaire (IPQ-R), which focuses on the individual perceptions of IBS, and questions regarding the health care use. In addition, sociodemographic data, medical history and the Rome IV criteria were assessed.

The questionnaire was embedded in a number of other questions which were not part of the presented project.

#### Illness perception questionnaire – revised (IPQ-R)

The German version of the IPQ-R [23] was used to assess the individual illness perception. The first version of the IPQ was published in 1996 [24], the revised version in 2002 [25]. Since then it is well-established and frequently utilised all over the world. The internal reliability of the IPQ-R subscales and test–retest reliability were reported to be satisfactory [23]. The German version of the IPQ-R [23] consists of 64 items for nine dimensions in three domains:

i) In the first domain, called “illness identity”, the clinical signs and symptoms are assessed (14 items), which are a.) experienced and b.) attributed as symptom of the illness (one dimension, called “identity”).
ii) The second domain (32 items, called “beliefs domain”) asks for illness beliefs and consists of seven clinical dimensions (“time course”, “consequence”, “personal control”, “cure control”, “coherence”, “cyclic occurrence”, and “emotional representation”). An overview of these seven IPQ-R dimensions and their interpretation are displayed in Table 1.
iii) In the third domain (18 items, called “causes domain”), the assumed causes for IBS were queried (one dimension, called “cause”). Answers were given on a five-point Likert-Scale (“strongly disagree” to “strongly agree”). The IPQ-R subscale “causes” cannot be aggregated to one score. Therefore, causes which were chosen as relevant from an item list (“agree” and “strongly agree”) of at least 50% and at least 75% of participants were reported.

**Table 1:** IPQ-R subscales from the domain “beliefs” and their interpretation

Dimensions of 2^nd^ domain (“beliefs”)	Interpretation
Time course	The higher the value, the more chronic the IBS.
Consequences	The higher the value, the more consequences of IBS.
Personal control	The higher the value, the better the personal control about IBS symptoms.
Treatment control	The higher the value, the better the treatment response.
Coherence	The higher the value, the more comprehensible the IBS.
Cyclic occurrence	The higher the value, the more periodic the occurrence of IBS.
Emotional representation	The higher the value, the more emotional strain caused by IBS.

The term “irritable bowel syndrome” was used instead of “illness” in the original questionnaire

In our study, the domain “illness identity” was waived for the following reason: IBS is conceptualized in different ways – some researchers and clinicians consider IBS as a somatisation disorder, e.g. according to the International Classification of Diseases (ICD-10) [26], some others label IBS as gut-brain axis disorder and any symptoms of somatization, that may be present, as additional psychological comorbidity [27]. There is no contradiction between these conceptualizations, but only different points of view on the same phenomenon.

However, the intrinsic proximity of the concept of somatisation to the concept of illness identity (which is described as “process of matching symptoms to an illness label” [25]) implies, that it is not reasonable to interpret this subscale in a study focusing on IBS as a disease which is considered to be a somatization disorder itself.

This approach has not only been chosen by colleagues [28], but it is also legitimized by the authors of the IPQ-R and their ongoing encouragement to adjust the scale to the research setting and the condition investigated [25]. Also based on this reasoning, the term “illness” in the IPQ-R was replaced by “irritable bowel syndrome” in our study.

### Use of the health care system

In absence of a suitable validated instrument, a self-developed questionnaire was used. Doing so the term “health care use” was operationalised using the following four criteria and questions:

i) Frequency of doctor visits because of IBS during the last year:
  Study participants with up to five appointments were considered as “normal utilisers”, those with more than five as “high utilisers”. In the absence of an established classification this cut off was chosen given the fact that up to five appointments may be needed for a full diagnostic assessment of IBS complaints by family doctors, gastroenterologists, gynaecologists, and/or psychologists according to the current German medical guideline [29]. A further question asked for the specialisation of the consulted physicians.
ii) Performed examinations: Participants were asked, whether they had medical examinations due to the (suspected) IBS, and if yes, which ones.
iii) Medication: Participants were asked, if they took medication because of IBS, and if yes, which ones (prescribed and over-the-counter).
iv) Treatment approaches with lacking or weak evidence for IBS: Participants were asked, if they made use of alternative methods and if yes, which ones.

For questions ii) – iv) multiple predefined answers and the use of a free description field were possible.

### Sociodemographics and medical history

Further information was collected: An ultra-short version of the Patient Health Questionnaire (PHQ-4) was used. It is a self-rating 4-item screening tool for anxiety and depression, resulting in a total score ranging from 0 to 12 points [30].

In addition, we asked for age and gender, the highest school-leaving qualification, the highest professional qualification, as well as gastrointestinal and mental comorbidities (categorical question (yes/no) regarding: Crohn’s Disease, ulcerative colitis, stomach or bowel cancer, diverticular disease, adhesions, hernias, celiac disease, malabsorption of fructose or lactose, food allergies, depression, anxiety or panic disorder, somatisation disorder, eating disorder and an additional description field).

### Rome IV criteria as validation of the diagnosis of IBS

For confirmation of the IBS diagnosis, the Rome IV questionnaire for IBS was used [22, 31]. IBS is defined as having “recurrent abdominal pain, on average at least one day per week in the last three months, associated with two or more of the following criteria: 1. Related to defecation, 2. Associated with a change in frequency of stool, and 3. Associated with a change in form (appearance) of stool” [31]. Changes in stool frequency, consistency and relationship to defecation were counted as “yes”, if the symptoms were present in at least 30% of time. All criteria had to be fulfilled for the last three months, and the symptom onset had to be at least six months ago.

### Statistical analyses

A two-step approach was chosen for the statistical analysis:

In a first step, the study population and its health care use were analysed (research question 1) using descriptive methods. Subgroup comparisons regarding frequency of doctor visits, medication intake, and use of treatment approaches with lacking or weak evidence for IBS (research question 2) were performed after checking the Gaussian distribution of data using the Kolmogorov–Smirnov test. Because of skewed data distribution and different subgroup sizes, all comparisons used non-parametric methods (Mann Whitney U-test). The corresponding group size (n) is reported for each analysis. Pearson correlation coefficient (Pearson’s r) was chosen as effect size. As a rule of thumb, it was considered as small if r ≥ 0.1 and < 0.3, moderate if r ≥ 0.3 and < 0.5, and large if r ≥ 0.5 [32, 33]. Because of the lack of scientific literature regarding the relationship of illness perception and health care use in IBS affected persons, effects of every size were considered as “relevant”. Its implications are discussed below.

In a second step, a multivariable regression model was performed focussing on prediction of frequency of doctor appointments and the use of treatment approaches with lacking or weak evidence as dependent variable (research question 3). Variables which turned out to be significantly different between the investigated subgroups in step one of our analysis were considered as predictors. Age, gender and anxiety/depression (PHQ-4) were forced into the model to control for the effects of these context variables (Regression model 1a) and to enhance external validity of our results.

To control for the influence of the selection of the study sample, the main regression model was done twice: in a second model (Regression model 1b) the factor “Rome IV status” was added as covariable.

**Fig. 1:**
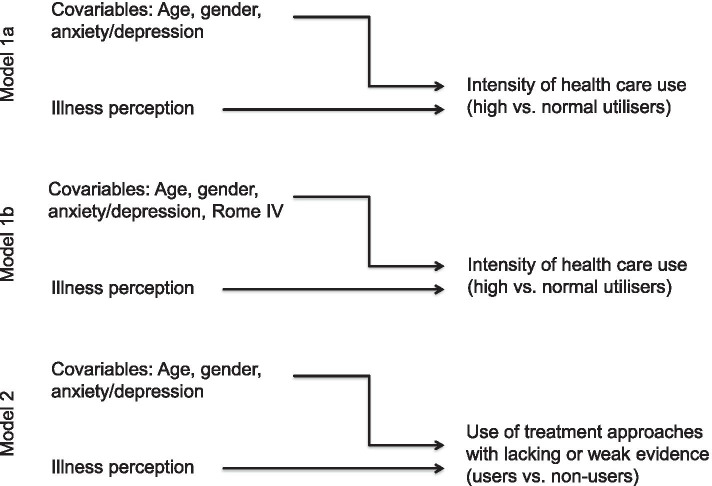
Methodological strategy (Logistic regression models)

Prediction of the use of treatment approaches with lacking or weak evidence was addressed with the help of a further logistic regression model (Regression model 2) with variables significantly different between the subgroups in step 1 as predictors. Similarly like in the regression models 1a and 1b, age, gender, and PHQ-4 status were forced into the model. An overview of the regression models is given in Fig. 1.

Multicollinearity within the potential predictors was tested using variance inflation factor (had to be ≤ 5) and conditions index (had to be ≤ 30). The Nagelkerke R^2^ was interpreted as explained variance, and the Hosmer–Lemeshow test was used to evaluate the goodness of fit. Results were reported as OR with corresponding 95% confidence interval (95% CI). According to Chen et al. [33] OR was interpreted as effect size. An OR between ~ 1.5 and ~ 2.7 was considered as small, an OR between ~ 2.7 and ~ 4.7 as intermediate, and over 4.7 as large effect.

The sample size was calculated under the assumption, that regression analysis needs ten to 15 cases per predicting variable and group. Therefore, an absolute minimum of 50 participants was required.

In all analyses the level of significance was set to be α = 0.05. Excel 2019 was used to calculate the effect size r and for the descriptive analysis of the third IPQ-R domain, all other statistical analyses were performed using SPSS version 26.0.0.0.

## Results

### Characteristics of the study population

The unadjusted total sample consisted of N = 528 participants, of which 15 were excluded because of lacking consent (“consent item” was not ticked, n = 1), and missing information about age (considered as not being of adult age, n = 14) resulting in an adjusted total sample of N = 513 participants.

The sample was predominantly female, with a median age of 32 years. Two-third of the participants had a university-entrance diploma and over 40% a university degree. Just over one half had a history of a gastroenterological or mental illness, with depression (17.3%), anxiety (16.9%), and abdominal adhesions (6.3% of the total sample) being the most frequent conditions. Crohn’s Disease (1.2%) and ulcerative colitis (0.2%) were rare. Nearly two third of the participants fulfilled Rome IV criteria. Details can be found in Table 2 and in Additional file 1.

**Table 2:** Sociodemographics

**Variable**	**Unit / Category**	**Valid cases**	
Age	years/median (range)	513	32 (19–77)
Gender	% (n) / female	498	82.1 (409)
Highest school-leaving qualification			
Intermediate school leaving diploma	% (n)	508	20.7 (105)
Advanced technical college certificate	% (n)	508	10.6 (54)
University-entrance diploma	% (n)	508	66.3 (337)
Other graduation	% (n)	508	2.4 (12)
Highest professional qualification			
In training	% (n)	510	19.6 (100)
Apprenticeship	% (n)	510	22.7 (116)
Technical college / professional school	% (n)	510	9.2 (47)
University degree (incl. university of applied sciences)	% (n)	510	42.5 (217)
No training qualification, not in training	% (n)	510	1.8 (9)
Other qualification	% (n)	510	4.1 (21)

The vast majority consulted a physician because of IBS symptoms (89.3%), most of them between one and five times during the last year (53.6%). 71.7% of the total sample were seen by a general practitioner (GP) as well as a medical specialist, 4.5% by a medical specialist only. The consulted specialists were predominantly in internal medicine (85.4%) and gynaecology (41.1% of females). However, many participants indicated “other medical specialists” as listed in our questionnaire and named “gastroenterologist”, obviously not knowing that this is a subspecialisation of internal medicine.

Over one third (35.7%) had six or more doctor visits due to IBS during the preceding year and was classified as "high utilisers", which implies one doctor visit every eight weeks on average; 6.6% had more than twenty doctor visits in the past year. The majority of patients, who consulted a physician, had diagnostic tests performed due to IBS. Almost 80% had a blood test, a smaller proportion had a stool sample analysed (72.1%) and had physical examination (63.7%). Gynaecological examinations were performed in one third of women only. Gastroscopy was carried out in 65.3%, colonoscopy in 73.7% of cases. Over 80% of the participants took at least one medication because of IBS. The most prevalent ones were anti-diarrhoea drugs (51.8%), proton pump inhibitors (44.5%), and non-steroidal antirheumatics (NSAR) (29.4%).

Treatment approaches with lacking or weak evidence were used by more than half of the population (57.7%), with homeopathy most common (31.4%), followed by yoga (19.3%), meditation (17.5%) and Traditional Chinese Medicine (13.5%). Further details regarding health care use can be found in Additional file 2.

Asked to indicate what they considered as possible causes of her or his illness, the items “stress” (84.2%), “diet” (53.5%) and “emotional state” (55.6%) gained broad approval.

### Health care use

#### Group comparisons

Comparing patients with high versus normal frequency of doctor appointments, the consequences of IBS on the IPQ-R subscales were rated as more serious in patients with more doctor contacts. This effect was of moderate size. Also emotional strain caused by IBS was higher, and experience of personal and cure control was reduced in high utilisers, but the effect sizes were small (Table 3).

**Table 3:** Illness perception: high vs. normal utilisers

**Subscale/Dimension**	**high utilisers**		**normal utilisers**					
	**n**	**Median**	**n**	**Median**	**U**	**Z**	**p**	**r**
Time course	173	22.00	311	21.00	23,806.50	-1.731	0.084	0.08
Consequences	168	20.00	308	18.00	15,947.50	-6.945	< 0.001	0.32
Personal control	175	11.00	312	12.00	22,647.50	-3.134	0.002	0.14
Cure control	174	9.00	307	10.00	22,797.50	-2.682	0.007	0.12
Coherence	174	12.00	303	12.00	25,125.50	-0.855	0.393	0.04
Cyclic occurrence	173	14.00	311	14.00	26,568.50	-0.228	0.82	0.01
Emotional representation	171	20.00	310	18.00	18,157.00	-5.737	< 0.001	0.26

In the illness perception questionnaire revised, the term “irritable bowel syndrome” was used instead of “illness” in the original questionnaire. r ≥ 0.1 and < 0.3  ⇒ small effect size, r ≥ 0.3 and < 0.5 ⇒ moderate effect size, r ≥ 0.5 ⇒ large effect size [32, 33]

Differences in illness perception were also found comparing participants taking medication due to IBS with those who indicated not to do so (Table 4). Medication users perceived significantly more consequences and less cure control and were more strained emotionally by IBS. However, the effect sizes were small.

**Table 4:** Illness perception: medication users vs. non-users

**Subscale/Dimension**	**medication users**		**medication non-users**					
	**n**	**Median**	**n**	**Median**	**U**	**Z**	**p**	**r**
Time course	420	21.00	68	21.00	13,437.50	-0.786	0.432	0.04
Consequences	415	19.00	71	17.00	10,595.00	-3.797	< 0.001	0.17
Personal control	425	11.00	73	12.00	14,376.00	-1.004	0.315	0.04
Cure control	420	10.00	71	11.00	12,692.50	-2.014	0.044	0.09
Coherence	420	12.00	68	13.00	13,526.00	-0.701	0.483	0.03
Cyclic occurrence	422	14.00	72	15.00	14,432.00	-0.686	0.493	0.03
Emotional representation	423	19.00	69	17.00	11,557.00	-2.781	0.005	0.13

In the illness perception questionnaire revised, the term “irritable bowel syndrome” was used instead of “illness” in the original questionnaire. r ≥ 0.1 and < 0.3 ⇒ small effect size, r ≥ 0.3 and < 0.5 ⇒ moderate effect size, r ≥ 0.5 ⇒ large effect size [32, 33]

Splitting the sample in users and non-users of treatment approaches with lacking or weak evidence, only the dimension “consequences” was found to be significantly different, yet with small effect size (Table 5).

**Table 5:** Illness perception: users vs. non-users of treatment approaches with weak or lack of evidence

**Subscale/Dimension**	**users**		**non-users**					
	**n**	**Median**	**n**	**Median**	**U**	**Z**	**p**	**r**
Time course	286	21.00	208	21.00	29,322.50	-0.271	0.787	0.01
Consequences	283	19.00	208	18.00	21,691.00	-5.000	< 0.001	0.23
Personal control	290	11.00	214	11.50	30,665.00	-0.227	0.821	0.01
Cure control	285	10.00	212	10.00	28,676.50	-0.973	0.331	0.04
Coherence	291	13.00	203	12.00	28,279.00	-0.808	0.419	0.04
Cyclic occurrence	287	14.00	213	14.00	28,995.50	-0.993	0.321	0.04
Emotional representation	285	19.00	213	19.00	27,433.50	-1.842	0.065	0.08

In the illness perception questionnaire revised, the term “irritable bowel syndrome” was used instead of “illness” in the original questionnaire. r ≥ 0.1 and < 0.3 ⇒ small effect size, r ≥ 0.3 and < 0.5 ⇒ moderate effect size, r ≥ 0.5 ⇒ large effect size [32, 33]

Comparing participants who fulfil the Rome IV criteria with those who do not, the Rome IV positive subgroup perceived their disease as more chronic, its appearance as more periodic, with more consequences in daily life, reduced personal and cure control, and impaired emotional representation of IBS (Table 6); however, the effect size of these differences were small.

**Table 6:** Illness perception: Participants fulfilling Rome IV criteria vs. not fulfilling

**Subscale/Dimension**	**Rome** IV **positive**		**Rome** IV **negative**					
	n	Median	n	Median	U	Z	p	r
Time course	317	22.00	177	21.00	23,163.50	-3.234	0.001	0.15
Consequences	315	19.00	176	18.00	22,804.50	-3.272	0.001	0.15
Personal control	319	11.00	185	12.00	24,742.50	-3.035	0.002	0.14
Cure control	316	9.00	181	11.00	22,457.50	-4.003	< 0.001	0.18
Coherence	315	12.00	179	13.00	25,697.50	-1.640	0.101	0.07
Cyclic occurrence	316	15.00	184	14.00	24,751.00	-2.802	0.005	0.13
Emotional representation	318	20.00	180	17.50	21,954.50	-4.333	< 0.001	0.19

In the illness perception questionnaire revised, the term “irritable bowel syndrome” was used instead of “illness” in the original questionnaire. r ≥ 0.1 and < 0.3 ⇒ small effect size, r ≥ 0.3 and < 0.5 ⇒ moderate effect size, r ≥ 0.5 ⇒ large effect size [32, 33]

#### Regression model

Prediction of frequency of doctor visits:

Substantial multicollinearity could be excluded (variance inflation factor ≤ 5, conditions index ≤ 30, data not shown), and modelling was satisfying (Hosmer–Lemeshow test, Chi^2^ = 13.322, df = 8, p = 0.104) with 16.9% of variance was explained (Nagelkerke R^2^ = 0.169). The regression model revealed age and the IPQ-R subscale “consequences” as predictors for the frequency of health care use as measured in this study. Every year of age reduced the odds for an intensive health care use (OR 0.980; 95%CI: 0.962–0.998; p = 0.027), and every point on the IPQ-R scale “consequences” increased the odds for an intensive health care use (OR 1.189; 95%CI: 1.100–1.284; p ≤ 0.001). The other variables (age, gender, PHQ4, and the IPQ-R-scales “emotional representation “, “personal control”, and “cure control”) lost significance. Both effect sizes were small. Statistical details can be found in Additional file 3. Adding the variable „Rome IV “ into the model did not change the results. (Regression model 1b, Fig. 1, details in Additional file 4).

Prediction of the use of treatment approaches with lacking or weak evidence:

We found no substantial multicollinearity (variance inflation factor ≤ 5, conditions index ≤ 30, data not shown). Hosmer–Lemeshow test showed satisfying goodness of fit (Chi^2^ = 12.545, df = 8, p = 0.129), and 9.3% of variance was explained (Nagelkerke R^2^ = 0.093). Female gender was found to be an independent predictor of the use of treatment approaches with lacking evidence (reference category male: OR 0.537; 95%CI: 0.327–0.881; p = 0.014) and the more consequences the patients felt, the higher the odds to use these methods (OR 1.155; 95%CI: 1.091–1.223; p ≤ 0.001). Both effects were of small size. Statistical details can be found in Additional file 5.

## Discussion

In this study we investigated the illness representation in IBS affected individuals, different aspects of their use of the health care system, and the relation between both. The perceived consequences and the emotional representation as subdomains of the illness perception seemed to be of primary relevance. Cure and personal control played a minor role. The perceived consequences predicted the use of the conventional health care system, as well as the use of treatment approaches with lacking or weak evidence regarding effectiveness in IBS. 

### Study population and characteristics of the sample

In accordance with the well-established association of IBS and female gender [1], the vast majority of the participants of our study were women. The median age was surprisingly low in comparison to that one of IBS-affected participants in other population-based studies [34]. This might be caused by our recruitment strategy via study announcements mainly in cities with large universities and partly using mailing lists of the university. The recruitment strategy might also contribute to our finding, since the proportion of participants with a university entry diploma or university degree was high compared to the general population in Germany [35], in line with findings that a low educational level may act as barrier for study participation [36]. However, an analysis of nonresponders was not possible due to the completely anonymous recruitment.

Almost two third of the participants indicated the presence of a mental or gastrointestinal diagnosis, which is in an expectable range [1]. About 20% reported a history of depression, a similar proportion a history of anxiety. Multiple answers were possible. The prevalence of depression and anxiety was lower than known from the literature: A recently published analysis based on routine data from a German health insurance plan found 58% of patients with IBS with “neurotic, stress-related and somatoform disorders”, and 42% with “affective disorders” (mainly depression) [37]. The same study group reported 45% with at least one further diagnosis affecting oesophagus, stomach and/or duodenum [37].

About two third of the study participants met the Rome IV criteria. This was less than expected and might be due to the fact that Rome IV is more restrictive than Rome III, resulting in a reduction of up to 50% of diagnosed cases [38]. However, this gap between the illness representation of the individual (“being affected with IBS”) and the diagnostic Gold standard (“fulfilling Rome IV criteria”), as well as the gap between the Rome III and Rome IV criteria underline the relevance of our research question, the individual conceptualisation of IBS.

Participants fulfilling the Rome IV criteria reported IBS to be more often chronic and recurrent, with more consequences in daily life, and with less cure and personal control than patients not meeting Rome IV criteria. Additionally, they indicated a higher emotional strain by IBS. With regard to illness perception, it is noticeable that the differences between Rome IV positive and negative study participants were small, but applied to almost every subscale of the IPQ-R. This may be seen as indicating consistently more dysfunctional illness perception in individuals fulfilling the Rome IV criteria as compared to their counterparts and it raises the question, whether the perception of the symptoms initiates the dysfunctional illness representation, or the other way around. Both – the gastrointestinal symptoms influence the illness representation or the representation influences the symptom perception – would be thinkable. Also, a bidirectional relationship with the development of a vicious circle between dysfunctional illness representation and perceived symptom severity would be possible. However, these hypotheses need further research activity and empirical validation.

### Health care use

Health care use was operationalised using four aspects: The frequency of doctor visits, the examinations carried out, the intake of medication, and the use of treatments with weak or lacking evidence for effectiveness in IBS treatment. This operationalisation is random to some extent, however it is consistent with the German health care system, in which the individual patient is free in the choice of the treating doctor and institution, and the access to health care is commonly without regulation within the statutory health care system. [39].

### Doctor visits, examinations, and medication

In our sample nine out of ten patients had seen a health care professional during the last year before the survey, which was high in comparison to other studies: An Australian working group found 51.3% of IBS affected community subjects seeking conventional care due to bowel problems during the last year [40]. More than one third of our sample was classified as “high utiliser” with six or more doctor visits due to IBS during the last year. This classification may be arbitrary to some extent, however we consider it as a suitable and rather conservative approach, as we will specify in detail below. A recently published French study [41] based on 30.000 records from the national health data system examined health care use five years before and one year after a hospital admission due to IBS and found > 80% of patients meeting their family doctor at least three times per year independent of the time (before/after admission). The authors also reported high rates of gastroenterological visits (approximately 80% of patients within the year before hospital admission), as we did (85.4% consulted an internal medicine specialist). However, our question was not restricted to the last year, and the investigated samples were different (hospital patients vs. an unselected sample in our case). Both studies found a more intensive health care use in comparison to an American study group, which reported 50.1% of participants visiting a gastroenterologist in a two years’ time period around the IBS diagnosis [42]. Moreover, the American researchers found a considerable heterogeneity between different US states. This underlines the role of insurance status as enabling factor in the sense of the Andersen model [4] as described in the introduction section.

Predicting high frequency of doctor visits from the individual disease representation via a logistic regression analysis, we found age and the perceived consequences of IBS as key factors. It is conceivable, that growing older with IBS may go along with better working or adjusted coping strategies, but this hypothesis could not be verified with our dataset because of lacking information about the duration of the disease.

The significant effect of the perceived IBS consequences in our regression model might be understood as encouragement for caregivers to focus more on individual functional capability in clinical practice, instead of the number or the intensity of the reported symptoms. This approach is already adopted in the German medical Guideline [29], which defines IBS not only as positive diagnosis with a set of symptoms, but also requires health care seeking and/or the fact, that the patient is worried about the symptoms and experiences an impairment of his or her quality of life.

The effect of the perceived consequences on health care use seemed to be independent of mental and sociodemographic aspects (PHQ-4, age, gender), but literature regarding the relationship of mental disorders and/or personality traits and health seeking behaviour is conflicting: Some researchers found associations between personality traits, mental and/or psychosocial factors and health care use [7, 43] – others did not [6].

Adding the variable „Rome IV “ in a further regression model (model 1b), the analysis yielded similar results than model 1a. This underlines the relevance of the assessment of subjective consequences in daily life (as assessed by e.g. IPQ-R) instead of an assessment of “pure” symptomatology (e.g. Rome IV) or focusing on symptom severity only. This finding is also in line with a study which examined the individuals’ complaint-related cognitions and the utilisation of the health care system: Van Dulmen and colleagues [21] conducted a two time-point follow-up study in the Netherlands and found attention paid by the professional to the patients’ disease representation to be associated with lowered health care use. Additionally, they reported somatic attribution of symptoms in patients not only related to intensive health care use, but also they proposed a link between somatic attribution in patients and referral behaviour of their GPs: “the more the GP referred to internal medicine, the more their patients attributed their abdominal complaints to a somatic abnormality” [21]. This underlines the need for clearly reflecting the indication for somatic diagnostics and for refraining from repeated investigations – not only with regards to increasing costs [44], but also because of its negative impact on the progression of functional diseases such as IBS. 

Not only over-, but also substantial underuse was found: In our study, only around one third of IBS affected women reported a gynaecological examination due to IBS, as it is recommended in the German guideline as first line diagnostic procedure [29]. In addition, further recommended examinations such as full blood count [29] were omitted in a significant proportion of patients, whereas other examinations such as gastroscopy and colonoscopy – useful, but not first line – were reported to be performed in over 2/3 and 3/4 of participants, respectively. Based on our data it would be daring and unreliable to label this as overuse. However, many participants in our sample reported colonoscopy due to IBS – which is associated with certain risks – but not risk-free urine examination. This may be due to a recall bias, but also limited adherence to guidelines would be a possible cause and has been described in IBS [45], as well as in other functional gastrointestinal conditions [44].

Limited guideline adherence could also contribute to the high proportion of medication use due to IBS: Almost one third of the study sample reported the use of NSAR because of IBS, which is against the recommendation of the German guideline [29]. It is reasonable to assume that many patients take NSAR without prescription and as over-the-counter medication, but it may also be understood as indicating an insufficient or ineffective provision of adequate therapy in the health care system.

### Treatment approaches with lacking or weak evidence regarding effectiveness in IBS

In our study, more than half of the patients indicated to use treatment approaches with lacking or weak evidence because of their IBS, and most of them were females. Female gender is also the strongest predictor in regression model 2. Both – the widespread use of evidence lacking methods, as well as the female predominance – are in line with other studies [46, 47]. The most prevalent method reported was homeopathy, followed by yoga and meditation. Whereas there were single studies which supported yoga as complementary therapy option in IBS [48], a recent published Cochrane review [49] found no sufficient evidence for a recommendation for homeopathy in IBS. An updated meta-analysis regarding mind–body interventions in IBS reported no effects of meditation as treatment [50], but considerable heterogeneity for meditation therapy, so no definitive recommendation was given.

Similarly to the regression models 1a und 1b with regard to conventional health care use, we found perceived consequences in daily life as second predicting factor for the use of evidence lacking methods; however the effect size was small.

The widespread use of evidence lacking methods in our sample may point towards a lack of trust in conventional medicine. Indeed we did not collect data regarding this question, but there is evidence suggesting that patients did not discuss the use of alternative medicine with their physician [46]. For health professionals it is important to know, if the patient uses over-the-counter medication and/or alternative medicine – not only because of the risk of medication interaction [47], but also to identify patient frustration with current treatments, which is an important reason to modify the current therapy.

### Limitations

To the best of our knowledge, there are only few studies examining the individual representation of IBS and the utilisation of the health care system. Doing so, not only the mainly public health insurance covered sector of conventional medicine was investigated, but also the use of treatment approaches with lacking or weak evidence for IBS treatment. The large database of over 500 participants is exceptional; however, this study has a few limitations:

First, it is important to clarify how to deal with the fact, that many of the reported effects in our study were of small or intermediate effect size – both in the group comparisons (Pearson’s r) and in the regression analysis (explained variance/Nagelkerke R^2^). However, considering the wide range of features predicting health care use in the Andersen model [4] (needs, predisposing and enabling factors) and having in mind that the individual illness perception reflects only one of these aspects, we did not expect large effect sizes. Indeed there is evidence for the influence of a positive modification of illness representation during a psychotherapeutical process on patients’ quality of life [51]. Therefore, we consider our findings as clinically relevant despite low effect sizes.

Second, a selection bias caused by our recruitment strategy seems likely: We assume an over-representation of academics and participants with higher mental strain, because this subgroup is more motivated to take part in a study like ours. This may have impact on our results.

Third, no objective validation of the diagnosis IBS or the information about health care use and other features was possible because of the nature as an internet-based questionnaire study. However, there is evidence that only about ten percent of patients with a positive diagnosis of IBS (according to Rome III) had a structural bowel lesion detectable with colonoscopy at a subsequent visit [52]. Applying Rome IV criteria this proportion is probably even lower, because of the more restrictive criteria [38]. Also, validity of self-reported medication and health care use seems to be satisfying [53].

Fourth, our classification of “high” versus “normal” with a cut off at five contacts to health professionals may be seen as arbitrary: We established above, that this cut off was chosen given the fact that up to five appointments may be needed for a full diagnostic assessment of IBS complaints according to the German medical guideline [29]. However, from a medical point of view, an optimally managed irritable bowel syndrome does not require five mandatory appointments per year. Therefore, our cut off can be considered as a rather conservative approach. This underlines the evidence for the postulated inappropriate health care use in patients suffering from IBS.

Fifth, due to the design of our study as cross-sectional study, causal relationships between illness perception and health care use cannot be drawn, thus our results have to be interpreted carefully and need further replication in longitudinal studies.

## Conclusions

In summary our study helped to improve the understanding of the illness perception in individuals with IBS. Two key messages seemed to be crucial: First, daily life consequences, perceived cure and personal control were found to be the most relevant clinical dimensions of the perception of IBS.

Second, an association of the individual disease perceptions and health care use seemed to be likely and has to be considered in clinical management of IBS.

### Implications for clinical practice and medical service

First, the key results of our study imply, that in clinical routine the focus should be on individual daily life functional capability rather than on pure symptomatology. This can be achieved with open questions and giving the patient the opportunity to talk about them [54].

Second, looking for medical advice and/or reduced quality of life should be part of the definition of IBS, as it is already the case in the German national guideline [29]. It should be avoided that people are labelled as “IBS patient” without personal strain. Previous reports of around 25% [6] up to 50% [55] of IBS-symptom affected people who never seek medical advice raise the question, whether this should be considered as “disease” and whether these individuals should be seen as patients, as it is done in many definitions.

Third, the conceptualisation of IBS as disease is largely based on the view of the professional who sees and treats the patient. This is especially evident in the ICD-10 [26], in which IBS is listed twice: In chapter F45.- (mental and behavioural disorders), mainly used by psychiatrists, as well as in chapter K58.- (diseases of the digestive system), commonly applied by gastroenterologists. Interestingly the diagnosis F45.32 “somatoform autonomic dysfunction of the lower gastrointestinal tract” [26] can only be coded, if “disease anxiety” and/or “striking illness behaviour” is present, in contrast to the diagnosis K58.-, which distinguishes between the IBS subtypes (K58.1 diarrhoea predominant, K58.2 constipation predominant, and K58.3 mixed).

To deal with such differential perception of IBS, interdisciplinary collaboration between health care professionals is needed: There is evidence for a better outcome of collaborative care [56] not only between gastroenterologists and psychologists [57], but also other medical specialists [58], and dieticians [59].

Optimally, this interdisciplinary collaboration should be coordinated by a primary care physician , as it could be shown that the coordination of care was related with a protection against oversupply [60], even if related with lowered patients’ satisfaction, but also with a higher health care quality, and a reduction in health care use and costs [61].

## Supplementary Information

Additional file 1 Information regarding the medical history of participants.

Additional file 2 Information regarding the health care use of participants.

Additional file 3 Statistic details regarding logistic regression model 1a: prediction of intensive utilisation of the health care system.

Additional file 4 Statistic details regarding logistic regression model 1b: prediction of intensive utilisation of the health care system (Rome IV variable forced into the model).

Additional file 5 Statistic details regarding logistic regression model 2: prediction of the use of treatment approaches with lacking or weak evidence for effectiveness in IBS.

## Data Availability

The datasets generated and analysed during the current study are not available. Public availability of data and materials is not covered by the given participation consent and the corresponding ethic approval. In case data is required further, please contact the corresponding author.

## Abbreviations

e.g.: (Exempli gratia) for example
GP: General practitioner
IBS: Irritable bowel syndrome
ICD-10: International Classification of Diseases
IPQ(-R): Illness perception questionnaire (revised)
NSAR: Non-steroidal antirheumatic drugs
OR: Odds ratio
PHQ-4: Patient Health Questionnaire 4
vs.: Versus
