# Supplementary material for: Illness perception and health care use in individuals with irritable bowel syndrome: results from an online survey
Source: BMC Fam Pract. 2021 Jul 19;22:154. doi: 10.1186/s12875-021-01499-5 (PMC8287688; doi:10.1186/s12875-021-01499-5)
Supplement: Supplementary file 1 — Information regarding the medical history of participants. [file 12875_2021_1499_MOESM1_ESM.docx]

Additional file 1: Medical history

| **Variable** | **Unit / Category** | **Valid cases** |  |
| --- | --- | --- | --- |
| History of gastroenterological or mental illness* | % (n) | 510 | 60.0 (306) |
| Adhesions | % (n) | 509 | 6.3 (32) |
| Celiac disease | % (n) | 509 | 1.2 (6) |
| Crohn’s Disease | % (n) | 509 | 1.2 (6) |
| Ulcerative colitis | % (n) | 509 | 0.2(1) |
| Depression | % (n) | 509 | 17.3 (88) |
| Anxiety | % (n) | 509 | 16.9 (86) |
| Rome criteria (current) | % (n) / positive | 513 | 63.4 (325) |

* multiple answers possible
