# Supplementary material for: Illness perception and health care use in individuals with irritable bowel syndrome: results from an online survey
Source: BMC Fam Pract. 2021 Jul 19;22:154. doi: 10.1186/s12875-021-01499-5 (PMC8287688; doi:10.1186/s12875-021-01499-5)
Supplement: Supplementary file 2 — Information regarding the health care use of participants. [file 12875_2021_1499_MOESM2_ESM.docx]

Additional file 2: Health care use

| **Variable** | **Unit / Category** | **Valid cases** |  |
| --- | --- | --- | --- |
| Doctor visits |  | | |
| *None* | % (n) | 513 | 2.9 (15) |
| *Only general practitioner* | % (n) | 513 | 20.9 (107) |
| *Only medical specialist* | % (n) | 513 | 4.5 (23) |
| *General practitioner and medical specialist* | % (n) | 513 | 71.7 (368) |
| Consulted medical specialist* |  | | |
| *Internal medicine* | % (n) | 391 | 85.4 (334) |
| *Gynaecology* | only females: % (n) | 309 | 41.1 (127) |
| *Urology* | % (n) | 391 | 6.6 (26) |
| *Neurology* | % (n) | 391 | 10.0 (39) |
| *Psychosomatic medicine* | % (n) | 391 | 16.9 (66) |
| *Psychotherapy (medical and psychological)* | % (n) | 391 | 25.1 (98) |
| *Orthopedy* | % (n) | 391 | 10.5 (41) |
| *Anesthesia / pain therapy* | % (n) | 391 | 3.3 (13) |
| *Others* | % (n) | 391 | 39.4 (154) |
| Frequency of doctor visits during the last year |  | | |
| *None* | % (n) | 496 | 10.7 (53) |
| *1 up to 5* | % (n) | 496 | 53.6 (266) |
| *6 up to 10* | % (n) | 496 | 21.2 (105) |
| *11 up to 20* | % (n) | 496 | 8.3 (41) |
| *More than 20* | % (n) | 496 | 6.3 (31) |
| Examinations* |  | | |
| *None* | % (n) | 498 | 3.6 (18) |
| *Physical examination* | % (n) | 498 | 63.7 (317) |
| *Blood test* | % (n) | 498 | 78.7 (392) |
| *Urine examination* | % (n) | 498 | 35.9 (179) |
| *Stool examination* | % (n) | 498 | 72.1 (359) |
| *Sonography* | % (n) | 498 | 67.9 (338) |
| *X-ray abdomen* | % (n) | 498 | 11.6 (58) |
| *Colonoscopy* | % (n) | 498 | 73.7 (367) |
| *Gastroscopy* | % (n) | 498 | 65.3 (325) |
| *Gynaecological examination* | only females: % (n) | 397 | 35.0 (139) |
| *Respiratory examination* | % (n) | 498 | 40.4 (201) |
| *Food intolerance* | % (n) | 498 | 66.1 (329) |
| *Others* | % (n) | 498 | 9.8 (49) |
| Medication* |  | | |
| *None* | % (n) | 506 | 14.4 (73) |
| *Non-steroidal antirheumatics* | % (n) | 506 | 29.4 (149) |
| *Opioids* | % (n) | 506 | 3.2 (16) |
| *Antibiotics* | % (n) | 506 | 7.1 (36) |
| *Proton pump inhibitors* | % (n) | 506 | 44.5 (225) |
| *Anti-diarrhoea drugs* | % (n) | 506 | 51.8 (262) |
| *Purgative / evacuant / abstergent agent* | % (n) | 506 | 14.2 (72) |
| *Antidepressant drugs* | % (n) | 506 | 17.4 (88) |
| *Cortisone* | % (n) | 506 | 3.2 (16) |
| *Tranquilizer / sleep-inducing drugs* | % (n) | 506 | 18.6 (94) |
| *Others* | % (n) | 506 | 32.8 (166) |
| Use of treatment approaches  with lacking or weak evidence regarding effectiveness in IBS* |  | | |
| *None* | % (n) | 513 | 42.3 (217) |
| *Massage* | % (n) | 513 | 10.5 (54) |
| *Physiotherapy* | % (n) | 513 | 9.0 (46) |
| *Meditation* | % (n) | 513 | 17.5 (90) |
| *Yoga* | % (n) | 513 | 19.3 (99) |
| *TaiChi* | % (n) | 513 | 1.6 (8) |
| *Homeopathy* | % (n) | 513 | 31.4 (161) |
| *Traditional Chinese Medicine* | % (n) | 513 | 13.5 (69) |
| *Anthroposophical treatment* | % (n) | 513 | 3.3 (17) |
| *Spiritual healing* | % (n) | 513 | 1.6 (8) |
| *Others* | % (n) | 513 | 15.4 (79) |

* multiple answers possible
