# Supplementary material for: Illness perception and health care use in individuals with irritable bowel syndrome: results from an online survey
Source: BMC Fam Pract. 2021 Jul 19;22:154. doi: 10.1186/s12875-021-01499-5 (PMC8287688; doi:10.1186/s12875-021-01499-5)
Supplement: Supplementary file 3 — Statistic details regarding logistic regression model 1a: prediction of intensive utilisation of the health care system. [file 12875_2021_1499_MOESM3_ESM.docx]

Additional file 3: Logistic regression model 1a: prediction of intensive utilisation of the health care system

| Dependent variable: high (1) versus normal (0) utilisers  independent variables: gender (male=1), age, consequences (IPQ-R), emotional representation (IPQ-R), personal control (IPQ-R), cure control (IPQ-R), and PHQ-4. (METHOD=ENTER command)  Valid cases: n=425   \| **Omnibus Tests of Model Coefficients** \| \| \| \| \| \| --- \| --- \| --- \| --- \| --- \| \|  \| \| Chi-square \| df \| Sig. \| \| Step 1 \| Step \| 55.770 \| 7 \| .000 \| \| Block \| 55.770 \| 7 \| .000 \| \| Model \| 55.770 \| 7 \| .000 \|  - The designed model is an improvement over the baseline model.  \| **Model Summary** \| \| \| \| \| --- \| --- \| --- \| --- \| \| Step \| -2 Log likelihood \| Cox & Snell R Square \| Nagelkerke R Square \| \| 1 \| 499.635^a^ \| .123 \| .169 \| \| a. Estimation terminated at iteration number 5, because the parameter estimates changed by less than .001. \| \| \| \|  - Explained variance: 16.9%  \| **Hosmer and Lemeshow Test** \| \| \| \| \| --- \| --- \| --- \| --- \| \| Step \| Chi-Square \| df \| Sig. \| \| 1 \| 13.233 \| 8 \| .104 \|  - The model showed satisfying goodness of fit.  \| **Variables in the Equation** \| \| \| \| \| \| \| \| \| \| \| --- \| --- \| --- \| --- \| --- \| --- \| --- \| --- \| --- \| --- \| \|  \| \| Regression Coefficient B \| Standard Error \| Wald \| df \| Sig. \| Exp(B) \| 95% CI for EXP(B) \| \| \| Lower \| Upper \| \| Step 1^a^ \| Gender(1) \| .013 \| .284 \| .002 \| 1 \| .963 \| 1.013 \| .580 \| 1.770 \| \| Age \| -.020 \| .009 \| 4.880 \| 1 \| .027 \| .980 \| .962 \| .998 \| \| Consequences \| .173 \| .040 \| 19.130 \| 1 \| .000 \| 1.189 \| 1.100 \| 1.285 \| \| Emotional representation \| .034 \| .029 \| 1.379 \| 1 \| .240 \| 1.034 \| .978 \| 1.095 \| \| Personal control \| -.047 \| .040 \| 1.392 \| 1 \| .238 \| .954 \| .882 \| 1.032 \| \| Cure control \| .006 \| .042 \| .024 \| 1 \| .877 \| 1.006 \| .927 \| 1.092 \| \| PHQ-4 \| .019 \| .043 \| .200 \| 1 \| .655 \| 1.019 \| .937 \| 1.109 \| \| Constant \| -3.401 \| .964 \| 12.442 \| 1 \| .000 \| .033 \|  \|  \| \| a. Variables entered in step 1: gender, age, consequences, emotional representation, personal control, cure control, PHQ-4 \| \| \| \| \| \| \| \| \| \| |
| --- | --- | --- | --- | --- | --- | --- | --- | --- | --- | --- | --- | --- | --- | --- | --- | --- | --- | --- | --- | --- | --- | --- | --- | --- | --- | --- | --- | --- | --- | --- | --- | --- | --- | --- | --- | --- | --- | --- | --- | --- | --- | --- | --- | --- | --- | --- | --- | --- | --- | --- | --- | --- | --- | --- | --- | --- | --- | --- | --- | --- | --- | --- | --- | --- | --- | --- | --- | --- | --- | --- | --- | --- | --- | --- | --- | --- | --- | --- | --- | --- | --- | --- | --- | --- | --- | --- | --- | --- | --- | --- | --- | --- | --- | --- | --- | --- | --- | --- | --- | --- | --- | --- | --- | --- | --- | --- | --- | --- | --- | --- | --- | --- | --- | --- | --- | --- | --- | --- | --- | --- | --- | --- | --- | --- | --- | --- | --- | --- | --- | --- | --- | --- | --- | --- | --- | --- | --- | --- | --- | --- | --- | --- | --- | --- | --- | --- | --- | --- | --- | --- | --- | --- | --- | --- | --- | --- |
