# Supplementary material for: Illness perception and health care use in individuals with irritable bowel syndrome: results from an online survey
Source: BMC Fam Pract. 2021 Jul 19;22:154. doi: 10.1186/s12875-021-01499-5 (PMC8287688; doi:10.1186/s12875-021-01499-5)
Supplement: Supplementary file 4 — Statistic details regarding logistic regression model 1b: prediction of intensive utilisation of the health care system (Rome IV variable forced into the model). [file 12875_2021_1499_MOESM4_ESM.docx]

Additional file 4: Logistic regression model 1b: prediction of intensive utilisation of the health care system (Rome IV variable forced into the model)

| If the variable “Rome IV” is forced into the model, the main results stay similar:  Dependent variable: high (1) versus normal (0) utilisers  independent variables: age, gender (male=1), consequences (IPQ-R), emotional representation (IPQ-R), personal control (IPQ-R), cure control (IPQ-R), PHQ-4, and Rome IV (negative=1) (METHOD=ENTER command)  Valid cases: n=425   \| **Omnibus Tests of Model Coefficients** \| \| \| \| \| \| --- \| --- \| --- \| --- \| --- \| \|  \| \| Chi-square \| df \| Sig. \| \| Step 1 \| Step \| 56.122 \| 8 \| .000 \| \| Block \| 56.122 \| 8 \| .000 \| \| Model \| 56.122 \| 8 \| .000 \|  - The designed model is an improvement over the baseline model.  \| **Model Summary** \| \| \| \| \| --- \| --- \| --- \| --- \| \| Step \| -2 Log likelihood \| Cox & Snell R Square \| Nagelkerke R Square \| \| 1 \| 499.283^a^ \| .124 \| .170 \| \| a. Estimation terminated at iteration number 5, because the parameter estimates changed by less than .001. \| \| \| \|  - Explained variance: 17.0%  \| **Hosmer and Lemeshow Test** \| \| \| \| \| --- \| --- \| --- \| --- \| \| Step \| Chi-Square \| df \| Sig. \| \| 1 \| 13.582 \| 8 \| .093 \|  - The model showed satisfying goodness of fit.  \| **Variables in the Equation** \| \| \| \| \| \| \| \| \| \| \| --- \| --- \| --- \| --- \| --- \| --- \| --- \| --- \| --- \| --- \| \|  \| \| Regression Coefficient B \| Standard Error \| Wald \| df \| Sig. \| Exp(B) \| 95% CI for EXP(B) \| \| \| Lower \| Upper \| \| Step 1^a^ \| Gender(1) \| .027 \| .286 \| .009 \| 1 \| .926 \| 1.027 \| .587 \| 1.797 \| \| Age \| -.020 \| .009 \| 4.899 \| 1 \| .027 \| .980 \| .962 \| .998 \| \| Consequences \| .173 \| .040 \| 19.101 \| 1 \| .000 \| 1.189 \| 1.100 \| 1.284 \| \| Emotional representation \| .032 \| .029 \| 1.180 \| 1 \| .277 \| 1.032 \| .975 \| 1.093 \| \| Personal control \| -.047 \| .040 \| 1.349 \| 1 \| .245 \| .954 \| .882 \| 1.033 \| \| Cure control \| .008 \| .042 \| .041 \| 1 \| .840 \| 1.008 \| .929 \| 1.095 \| \| PHQ-4 \| .020 \| .043 \| .211 \| 1 \| .646 \| 1.020 \| .938 \| 1.109 \| \| Rome IV (1) \| -.139 \| .235 \| .351 \| 1 \| .553 \| .870 \| .549 \| 1.379 \| \| Constant \| -3.335 \| .971 \| 11.806 \| 1 \| .001 \| .036 \|  \|  \| \| a. Variables entered in step 1: gender, age, consequences, emotional representation, personal control, cure control, PHQ-4, Rome IV \| \| \| \| \| \| \| \| \| \| |
| --- | --- | --- | --- | --- | --- | --- | --- | --- | --- | --- | --- | --- | --- | --- | --- | --- | --- | --- | --- | --- | --- | --- | --- | --- | --- | --- | --- | --- | --- | --- | --- | --- | --- | --- | --- | --- | --- | --- | --- | --- | --- | --- | --- | --- | --- | --- | --- | --- | --- | --- | --- | --- | --- | --- | --- | --- | --- | --- | --- | --- | --- | --- | --- | --- | --- | --- | --- | --- | --- | --- | --- | --- | --- | --- | --- | --- | --- | --- | --- | --- | --- | --- | --- | --- | --- | --- | --- | --- | --- | --- | --- | --- | --- | --- | --- | --- | --- | --- | --- | --- | --- | --- | --- | --- | --- | --- | --- | --- | --- | --- | --- | --- | --- | --- | --- | --- | --- | --- | --- | --- | --- | --- | --- | --- | --- | --- | --- | --- | --- | --- | --- | --- | --- | --- | --- | --- | --- | --- | --- | --- | --- | --- | --- | --- | --- | --- | --- | --- | --- | --- | --- | --- | --- | --- | --- | --- | --- | --- | --- | --- | --- | --- | --- | --- | --- |
