# Supplementary material for: Illness perception and health care use in individuals with irritable bowel syndrome: results from an online survey
Source: BMC Fam Pract. 2021 Jul 19;22:154. doi: 10.1186/s12875-021-01499-5 (PMC8287688; doi:10.1186/s12875-021-01499-5)
Supplement: Supplementary file 5 — Statistic details regarding logistic regression model 2: prediction of the use of treatment approaches with lacking or weak evidence for effectiveness in IBS. [file 12875_2021_1499_MOESM5_ESM.docx]

Additional file 5: Logistic regression model 2: prediction of the use of treatment approaches with lacking or weak evidence for effectiveness in IBS

| Dependent variable: use (1) non-use (0)  independent variables: Gender (male=1), age, consequences (IPQ-R), PHQ-4  (METHOD=ENTER command)  Valid cases: n=466   \| **Omnibus Tests of Model Coefficients** \| \| \| \| \| \| --- \| --- \| --- \| --- \| --- \| \|  \| \| Chi-square \| df \| Sig. \| \| Step 1 \| Step \| 33.403 \| 4 \| .000 \| \| Block \| 33.403 \| 4 \| .000 \| \| Model \| 33.403 \| 4 \| .000 \|  - The designed model is an improvement over the baseline model.  \| **Model Summary** \| \| \| \| \| --- \| --- \| --- \| --- \| \| Step \| -2 Log likelihood \| Cox & Snell R Square \| Nagelkerke R Square \| \| 1 \| 599.493^a^ \| .069 \| .093 \| \| a. Estimation terminated at iteration number 4, because the parameter estimates changed by less than .001. \| \| \| \| \|  - Explained variance: 9.3%  \| **Hosmer and Lemeshow Test** \| \| \| \| \| --- \| --- \| --- \| --- \| \| Step \| Chi-square \| df \| Sig. \| \| 1 \| 12.545 \| 8 \| .129 \|  - The model showed satisfying goodness of fit.  \| **Variables in the Equation** \| \| \| \| \| \| \| \| \| \| \| --- \| --- \| --- \| --- \| --- \| --- \| --- \| --- \| --- \| --- \| \|  \| \| Regression Coefficient B \| Standard Error \| Wald \| df \| Sig. \| Exp(B) \| 95% CI for EXP(B) \| \| \| Lower \| Upper \| \| Schritt 1^a^ \| Gender(1) \| -.622 \| .252 \| 6.064 \| 1 \| .014 \| .537 \| .327 \| .881 \| \| Age \| -.006 \| .008 \| .626 \| 1 \| .429 \| .994 \| .979 \| 1.009 \| \| Consequences \| .144 \| .029 \| 24.305 \| 1 \| .000 \| 1.155 \| 1.091 \| 1.223 \| \| PHQ-4 \| -.066 \| .037 \| 3.215 \| 1 \| .073 \| .936 \| .871 \| 1.006 \| \| Constant \| -1.592 \| .532 \| 8.945 \| 1 \| .003 \| .204 \|  \|  \| \| a. Variables entered in step 1: gender, age, consequences, PHQ-4 \| \| \| \| \| \| \| \| \| \| |
| --- | --- | --- | --- | --- | --- | --- | --- | --- | --- | --- | --- | --- | --- | --- | --- | --- | --- | --- | --- | --- | --- | --- | --- | --- | --- | --- | --- | --- | --- | --- | --- | --- | --- | --- | --- | --- | --- | --- | --- | --- | --- | --- | --- | --- | --- | --- | --- | --- | --- | --- | --- | --- | --- | --- | --- | --- | --- | --- | --- | --- | --- | --- | --- | --- | --- | --- | --- | --- | --- | --- | --- | --- | --- | --- | --- | --- | --- | --- | --- | --- | --- | --- | --- | --- | --- | --- | --- | --- | --- | --- | --- | --- | --- | --- | --- | --- | --- | --- | --- | --- | --- | --- | --- | --- | --- | --- | --- | --- | --- | --- | --- | --- | --- | --- | --- | --- | --- | --- | --- | --- | --- | --- | --- | --- | --- | --- | --- | --- | --- | --- |
